# A Lung-Targeted Lipid Nanoparticle System Delivers miRNA to Suppress Colorectal Cancer Pulmonary Metastases

**DOI:** 10.3390/pharmaceutics18060660

**Published:** 2026-05-27

**Authors:** Yuxiang Gantai, Ziyan Yang, Yinshuang Chen, Mengxi Chen, Yu Hu, Tingwei Ye, Jiayu Xu, Shenyue Zhou, Yuanyuan Yu, Yan Chen, Mengmeng Wang, Weitao Zhang, Jianqing Ruan, Haiyang Zhang, Weipeng Wang

**Affiliations:** 1 College of Pharmaceutical Sciences, Soochow University, Suzhou 215123, China; 2 Department of Pharmacy, The Second Affiliated Hospital of Soochow University, Suzhou 215123, China

**Keywords:** LuT-LNP, AM22, miRNA, *PARP1*, colorectal cancer pulmonary metastasis

## Abstract

**Background:** Colorectal cancer (CRC) is the third most commonly diagnosed cancer worldwide, with more than 90% patients dying from metastasis due to limited treatment options. Although miRNA-based therapeutics represent a promising strategy, their clinical application has been hindered by poor stability *in vivo* and the lack of efficient organ-specific delivery systems. **Methods:** In this study, we developed a lung-targeted lipid nanoparticle (LuT-LNP) platform for the delivery of a chemically modified miRNA, AM22, which demonstrated enhanced tumor-suppressive activity. By replacing cholesterol and helper lipids with 1,2-dipalmitoyl-sn-glycero-3-phosphocholine (DPPC), the most abundant lipid in pulmonary surfactant, and systematically optimizing the ratios of ionizable and cationic lipids, we obtained a LuT-LNP formulation with superior lung tropism. **Results:** The resulting LuT-LNPs exhibited excellent stability, biocompatibility, and efficient encapsulation and protection of AM22. Both *in vitro* and *in vivo*, AM22-loaded LuT-LNP (AM22@LuT-LNP) significantly inhibited the proliferation and migration of CRC cells and markedly suppressed lung metastasis in a mouse model. Mechanistic studies revealed that AM22 acts by targeting *Poly (ADP-ribose) polymerase 1* (*PARP1*), inducing DNA damage, and inhibiting the epithelial-mesenchymal transition (EMT) process. **Conclusions:** These findings established a lung-targeted delivery platform for miRNA-based therapy, offering a promising strategy for the treatment of colorectal cancer pulmonary metastasis (CRPM).

## 1. Introduction

Colorectal cancer (CRC) ranks as the third most common cancer globally, with persistently high annual incidence and mortality rates worldwide [1,2]. Epidemiological trends show a shift toward earlier-onset CRC, marked by a notable increase in cases under the age of 50 and rising incidence in developed countries [3,4]. More than 90% of cancer patients die from tumor metastasis. In advanced CRC, distant metastasis, commonly to the liver, lungs, and/or bones, is a critical factor associated with poor prognosis. The lungs are the most frequent site of extrathoracic metastasis in CRC, accounting for over 10% of all metastatic cases [5]. Currently, surgical intervention is often no longer curative for patients with advanced metastatic disease. Conventional chemotherapies, such as fluorouracil and oxaliplatin, as well as monoclonal antibodies targeting epidermal growth factor receptor and vascular endothelial growth factor, show limited efficacy in metastatic settings [6]. While programmed cell death protein 1 inhibitors have demonstrated benefit, they are effective only in the subset of patients with high microsatellite instability or deficient mismatch repair [7,8], who represent fewer than 15% of all CRC cases [9,10]. Thus, there remains an urgent need to develop novel therapeutic strategies for metastatic CRC.

MicroRNA (miRNA) is a class of non-coding RNAs. The first miRNA was discovered in *Caenorhabditis elegans* by Ambros in 1993, heralding the beginning of non-coding RNA research [11,12]. miRNAs typically bind to the 3′-untranslated region (3′-UTR) of target mRNAs and suppress gene expression through translational inhibition or mRNA degradation [13]. Among them, miR-34a is an endogenous tumor-suppressive miRNA linked to the p53 pathway, capable of inhibiting tumor cell proliferation and migration and inducing apoptosis through multiple mechanisms [14]. A miR-34a mimic, MRX34, entered clinical trials but was discontinued due to immune-related adverse events [15]. In our previous work, we introduced single-nucleotide substitutions in the non-seed region of miR-34a and identified a more potent and safer derivative termed AM22 [16]. However, the activity of AM22 in metastatic tumors remained unexplored, and an efficient delivery system was still lacking.

The naked miRNA molecule poorly penetrates cell membranes and is highly unstable under physiological conditions, characterized by a short half-life, low serum stability, and susceptibility to nuclease degradation [17,18]. An effective delivery system is therefore essential to improve the bioavailability and therapeutic potential of oligonucleotide drugs at the target site. To date, a variety of delivery systems have been explored, including lipid nanoparticles (LNPs), viral vectors, polymers, and exosomes. Among these, LNPs offer lower immunogenicity than viral vectors, better biocompatibility than many polymers, and scalable manufacturing, and have been clinically validated. LNP thus represents the most extensively studied nanomaterial platform for nucleic acid delivery and has achieved clinical success. The first LNP-based siRNA drug was approved in 2018 [19], and LNP technology later enabled the rapid deployment of mRNA vaccines against COVID-19 [19]. LNPs can efficiently encapsulate negatively charged nucleic acid within a lipid bilayer, protect them from serum nucleases, enter target cells via endocytosis, and ultimately escape the endosome to release the nucleic acids into the cytoplasm, where they can exert their therapeutic function [20,21]. Conventional LNPs consist of four components including an ionizable lipid, a helper phospholipid, cholesterol, and a PEGylated lipid. However, standard LNPs tend to accumulate predominantly in the liver and spleen [22]. To redirect LNPs to extrahepatic organs such as the lungs, several strategies have been explored. Cheng et al. proposed the Selective Organ Targeting (SORT) approach, which incorporated a permanently cationic lipid to enhance lung tropism [23]. Su et al. removed cholesterol and helper phospholipids and found that a three-component LNP with increased cationic lipid content could significantly improve lung targeting [24]. Others have chemically modified cationic lipid structures to alter organ distribution [25]. Despite these advances, a safe, effective, and robust lung-targeted LNP system for nucleic acid delivery remains urgently needed to broaden the therapeutic application of oligonucleotide drugs.

In this study, we developed a novel lung-targeted LNP system through systematic screening for the delivery of AM22. By substituting cholesterol and helper phospholipids with 1,2-dipalmitoyl-sn-glycero-3-phosphocholine (DPPC), the most abundant lipid in pulmonary surfactant, and optimizing its ratio to ionizable and cationic lipids, we designed a quadruple-component lung-targeted lipid LNP termed LuT-LNP, which exhibited pronounced pulmonary tropism. The resulting formulation demonstrated excellent stability, biocompatibility, and efficient lung accumulation in both healthy mice and colorectal cancer pulmonary metastasis (CRPM) mouse model. Delivered via LuT-LNP, AM22 potently suppressed CRPM progression *in vitro* and *in vivo*. We further elucidated its mechanisms of action against tumor proliferation and migration, providing new insights for the development of miRNA-based antitumor therapies.

## 2. Methods and Materials

### 2.1. Materials

1,2-Dilinoleyloxy-3-dimethylaminopropane (DLin-MC3-DMA), 1,2-distearoyl-sn-glycero-3-phosphocholine (DSPC), and DPPC were purchased from AVT Pharmaceutical Tech Co., Ltd. (Shanghai, China). 1,2-Dimyristoyl-sn-glycerol-methoxypolyethylene glycol 2000 (DMG-PEG2000) was obtained from Yusi Pharmaceutical Technology Co., Ltd. (Chongqing, China). Cholesterol was supplied by Ruixi Biological Technology Co., Ltd. (Xi’an, China). 1,2-Dioleoyl-3-trimethylammonium-propane (chloride salt) (DOTAP) and DiR were purchased from MedChemExpress (Monmouth Junction, NJ, USA; Cat# HY-112754A and Cat# HY-D1048, respectively). Hoechst 33,342 and LysoTracker Green DND-26 were sourced from Yeasen Biotechnology Co., Ltd. (Shanghai, China). D-Luciferin sodium was acquired from Jingkangen Biomedical Co., Ltd. (Wuhan, China). Sodium acetate buffer, annealing buffer, normal saline, 30% acrylamide, and 10% sodium dodecyl sulfate were obtained from Solarbio Science & Technology Co., Ltd. (Beijing, China). RNAiso Plus and the BCA Protein Assay Kit were purchased from Takara Biomedical Technology Co., Ltd. (Beijing, China). The FastPure Plasmid Mini Kit, 2 × SYBR qPCR Master Mix, 100 bp DNA Ladder, and 6 × DNA loading buffer were from Novizan Biotechnology Co., Ltd. (Nanjing, China). RPMI-1640 medium was procured from Procell Life Science & Technology Co., Ltd. (Wuhan, China). and premium-grade fetal bovine serum (FBS) was from Ozfan Biotechnology Co., Ltd. (Nanjing, China).

### 2.2. Preparation of LNPs and AM22@LuT-LNPs

LNPs were prepared using the thin-film hydration method. Briefly, the ionizable lipid DLin-MC3-DMA, the permanently cationic lipid DOTAP, DPPC, and DMG-PEG2000 at various molar ratio (e.g., 32.8%:16.4%:49.3%:1.5% for LuT-LNPs) were dissolved in chloroform to form the organic phase. The solvent was evaporated under reduced pressure using a rotary evaporator to form a thin lipid film. The film was then hydrated with DEPC-treated water or phosphate buffered saline (PBS), followed by probe sonication using an ultrasonic processor (Sonxi Ultrasonic Instrument Co., Ltd., Shanghai, China) at 40% amplitude (80 W) for 10 min, with a pulse cycle of 3 s on and 2 s off, to obtain the LNP dispersion.

For preparation of AM22@LuT-LNPs, the resulting LuT-LNPs were mixed with AM22 at a nitrogen-to-phosphorus (N/P) ratio of 4:1. The mixture was homogenized by extrusion through a 0.4 μm polycarbonate membrane 20 times using an Avanti mini-extruder, followed by another 20 extrusions through a 0.2 μm polycarbonate membrane, yielding AM22@LuT-LNPs with a uniform particle size.

### 2.3. Characterization of AM22@LuT-LNPs

The stability of LuT-LNPs was evaluated by monitoring their physicochemical properties after storage in PBS at 4 °C for over two weeks. During this period, particle size, zeta potential, and polydispersity index (PDI) were measured by dynamic light scattering using a Zetasizer Nano ZS90 (Malvern Panalytical Ltd., Malvern, UK). The morphology of representative LuT-LNPs was examined using transmission electron microscopy (TEM).

The stability of AM22@LuT-LNPs against RNase was assessed by agarose gel electrophoresis. Naked AM22 and AM22@LuT-LNPs were incubated with RNase for 0, 0.5, 1, 2, and 4 h. A 2.0% agarose gel was prepared with 1 × TAE buffer and heated until the agarose was completely dissolved. Gelred (gelred:tae = 1:20,000 *v*/*v*) was added and mixed thoroughly. The samples were loaded and electrophoresed at 120 v for 30 min. Nucleic acid bands were visualized using a gel imaging system.

To evaluate the binding capability of the nanoparticles, an agarose gel retardation assay was performed. Naked AM22 and AM22@LuT-LNPs at N/P ratios of 1:1, 2:1, 4:1, 6:1, and 8:1 were analyzed on a 2% agarose gel containing TAE buffer and GelRed (1:20,000 *v*/*v*) at a constant voltage of 120 V. The gel was then imaged.

### 2.4. Hemolysis Assay

Fresh mouse blood was collected in an ethylenediaminetetraacetic acid-coated tube and centrifuged at 1000 rpm for 8 min to isolate red blood cells (RBCs). The RBCs were washed three times with 0.9% saline. Different concentrations of LuT-LNPs were added to the RBC suspension. Ultrapure water and PBS served as positive and negative controls, respectively. After incubation at 37 °C for 30 min, the samples were centrifuged at 1000 rpm for 8 min, and the absorbance of the supernatant was measured at 570 nm using a microplate reader. The hemolysis rate was calculated as follows:

Hemolysis rate (%) = (OD_sample_ − OD_negative_)/(OD_positive_ − OD_negative_) × 100%

### 2.5. Cell Culture

The human CRC cell line T84 and the human embryonic kidney cell line HEK-293T were obtained from the Cell Bank of the Chinese Academy of Sciences. The murine CRC cell line CT26-Luc was purchased from Fenghui Biotechnology Co., Ltd. (Wuhan, China). T84 and CT26-Luc cells were cultured in RPMI-1640 medium supplemented with 10% FBS. HEK-293T cells were maintained in DMEM containing 10% FBS. All cells were incubated at 37 °C in a humidified atmosphere with 5% CO_2_.

### 2.6. Cellular Uptake and Lysosome Escape Assay

T84 cells (5 × 10^4^) were seeded in a 12-well plate for 12 h and then treated with DiR-labeled LuT-LNPs. After incubation for 0.5 or 4 h, cells were washed twice with PBS and incubated with LysoTracker Green for 30 min to label lysosomes. Following another PBS wash, cells were stained with Hoechst 33,342 for 15 min and imaged by inverted fluorescent microscope.

### 2.7. Methyl Thiazolyl Tetrazolium (MTT) Assay

Cells were seeded in 96-well plates at 5 × 10^3^ cells per well and cultured overnight. The medium was replaced with fresh medium containing various concentrations of LuT-LNPs (0, 10, 20, 50, 100, and 200 μg/mL). After 72 h, 10 μL of MTT solution was added to each well and incubated at 37 °C for 1 h. The medium was then removed, and 150 μL of dimethyl sulfoxide was added to dissolve the formazan crystals. The plate was shaken at 800 rpm for 10 min, and absorbance was measured at 570 nm.

### 2.8. Cell Counting kit-8 (CCK-8) Assay

For proliferation analysis, 5 × 10^3^ cells per well (100 μL) were plated in 96-well plates. After culturing for the indicated time, 10 μL of CCK-8 reagent was added to each well. Following 72 h of incubation, absorbance was measured at 450 nm.

### 2.9. Wound Healing Assay

Cell migration was assessed using a scratch wound assay. Cells (8 × 10^6^) were seeded into 12-well plates and grown to 90% confluence. A sterile 200 μL pipette tip was used to create a scratch, and detached cells were removed by washing with PBS. Cells were then starved in medium containing 5% FBS. Images were taken at 0, 24, and 48 h using an inverted microscope, and the scratch area was quantified.

### 2.10. Transwell Assay

Cell migration was also evaluated using Transwell chambers. Cells (2 × 10^4^ in 200 μL medium with 1% FBS) were seeded into the upper chamber, while the lower chamber contained 600 μL medium with 20% FBS. After 24 h, cells remaining in the upper chamber were removed with a cotton swab. Cells that migrated to the lower surface were fixed with 4% paraformaldehyde, stained with 0.1% crystal violet, and counted under a microscope.

### 2.11. Quantitative Real-Time Polymerase Chain Reaction (qPCR)

Total RNA was extracted using an RNA extraction kit according to the manufacturer’s instructions. qPCR was performed with SYBR FAST universal qPCR Kit (Vazyme Biotech Co., Ltd., Nanjing, China), and relative expression was normalized using the 2^−ΔΔCt^ method. Primer sequences were listed in Table 1.

### 2.12. Western Blot Analysis

Cells were lysed in radioimmunoprecipitation assay buffer containing protease and phosphatase inhibitors. Protein concentration was determined using the BCA Protein Assay Kit (Takara Biomedical Technology Co., Ltd., Beijing, China). Samples were mixed with 1× loading buffer containing dithiothreitol, boiled at 95 °C for 10 min, separated on 10% sodium dodecyl sulfate-polyacrylamide gel electrophoresis (80 V for 30 min, then 120 V for 60 min), and transferred to a polyvinylidene fluoride membrane (300 mA, 90 min). The membrane was blocked with 5% skim milk in Tris-buffered saline with Tween 20 at 37 °C for 1 h, washed, and incubated with primary antibody overnight at 4 °C. After washing, the membrane was incubated with horseradish peroxidase-conjugated secondary antibody at room temperature for 1 h, washed again, and signals were detected using a ChemiDoc™ MP Imaging System (Bio-Rad, Hercules, CA, USA).

### 2.13. RNA Sequencing and Analysis

T84 cells were transfected with AM22@LuT-LNP or Ctrl@LuT-LNP. Total RNA was extracted, immediately snap-frozen in liquid nitrogen, and stored at −80 °C. RNA samples were then sent to Genewiz for eukaryotic transcriptome sequencing. Raw reads were processed, aligned to the reference genome, and differentially expressed genes (DGEs) were identified using thresholds of |log_2_ fold change| ≥ 1 and *p* < 0.05. Gene Ontology enrichment analysis and heatmap visualization were subsequently performed.

### 2.14. Dual-Luciferase Reporter Assay

Human *PARP1* promoter and its mutant pattern were respectively cloned into the pmirGLO basic vector. The wild-type (WT) sequence was 5′-AGG CUG GAG AGA GAU UCU GUU G-3′, and the mutant (MUT) sequence was 5′-UCC GAC CUC UCU CUA AGA CAA C-3′. T84 cells were seeded in 96-well plates (6 × 10^3^ cells per well) and transfected with the AM22 or control vector together with the pmirGLO-PARP1 promoter/mutant for 24–48 h. Firefly and Renilla luciferase activities were measured using the Dual-Lumi™ Luciferase Assay Kit (RG088S, Beyotime Biotechnology Co., Ltd., Shanghai, China) according to the manufacturer’s protocol.

### 2.15. Animal Studies

BALB/c mice (5–6 weeks old) were purchased from GemPharmatech Co., Ltd. (Nanjing, China) and acclimatized for one week before experiments. All animal procedures were approved by the Animal Protection and Utilization Committee of Soochow University and conducted in accordance with ARRIVE guidelines and the NIH Guide for the Care and Use of Laboratory Animals.

(1) *In vivo* distribution of LNPs

DiR dye was mixed with LNPs on a vortex mixer for 20 min, and free dye was removed by ultracentrifugation at 120,000× *g* for 40 min. Mice were intravenously injected with 100 μg of DiR-labeled LNPs. After 6 h, mice were euthanized, and major organs (heart, lung, liver, spleen, kidney) were collected for *ex vivo* fluorescence imaging using an IVIS system (PerkinElmer, Waltham, MA, USA).

(2) *In vivo* efficacy of AM22@LuT-LNPs against CRPM

A CRPM model was established by intravenous injection of 4.5 × 10^5^ CT26-Luc cells in 100 μL PBS into BALB/c mice. After five days, lung bioluminescence was confirmed by *in vivo* imaging. Mice were randomized into three groups (*n* = 6) based on bioluminescence intensity: Ctrl@LuT-LNP (negative control), 5-FU (positive control), and AM22@LuT-LNP (treatment). Ctrl@LuT-LNP and AM22@LuT-LNP were administered at 1 OD per mouse on days 1, 5, 9, and 13. 5-FU was injected at 20 mg/kg on days 1, 3, 5, 7, 9, 11, and 13. Bioluminescence was monitored periodically, and body weight was recorded every five days.

### 2.16. Statistical Analysis

Data are presented as mean ± standard deviation from at least three independent experiments. Differences between two groups were analyzed by Student’s *t*-test or unpaired *t*-test. Multiple-group comparisons were performed using one-way or two-way analysis of variance followed by Tukey’s post hoc test. All analyses were conducted with GraphPad Prism 10.1.2; *p* ≤ 0.05 was considered statistically significant, and *p* > 0.05 was considered not significant (ns).

## 3. Results

### 3.1. Development of LuT-LNP

**Figure 1:**
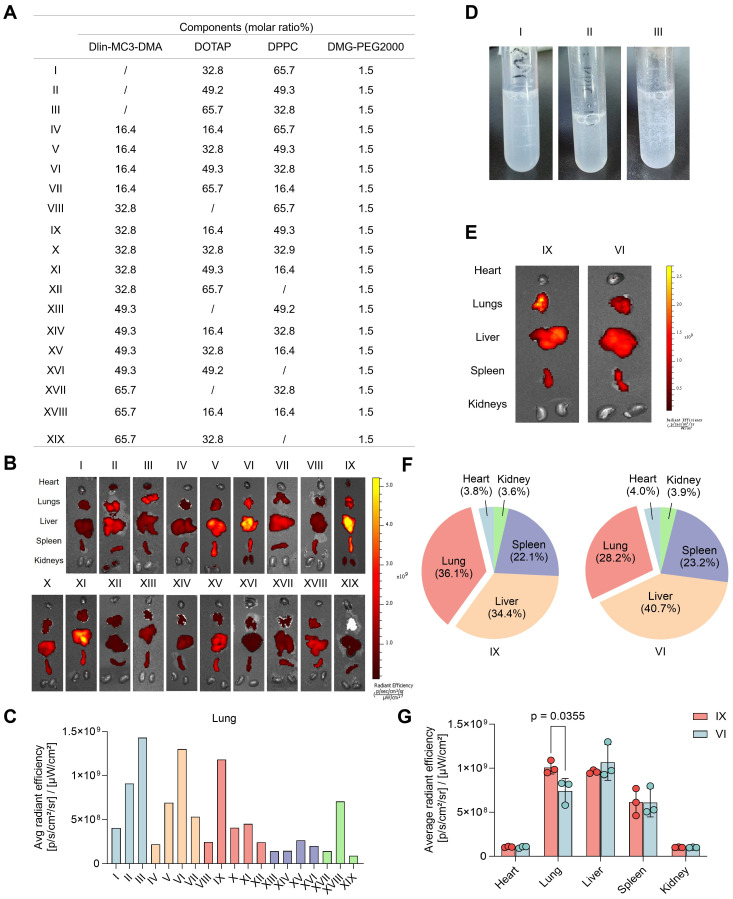
Screening of lipid formulations. (**A**) Composition table of LNP formulations for *in vivo* miRNA delivery validation. (**B**) *Ex vivo* fluorescence images of major organs after administration of different LNP formulations. (**C**) Quantification of fluorescence intensity in lung tissues. (**D**) Appearance of formulations I, II, and III showing aggregation after mixing with AM22. (**E**) *Ex vivo* fluorescence images comparing formulations IX and VI. (**F**) Organ distribution percentages of fluorescence intensity for formulations IX and VI. (**G**) Quantification of organ fluorescence intensity of formulations IX and VI.

Previous studies have established that modulating LNP lipid composition and ratios can alter organ tropism [24], and we sought to enhance pulmonary targeting by replacing cholesterol and DSPC with the pulmonary surfactant lipid DPPC. Across 19 formulations with fixed PEGylated lipid (1.5%) and varied DOTAP, DPPC, and Dlin-MC3-DMA content (0–65.7%; Figure 1A), initial *in vivo* bio-distribution revealed that binary DOTAP/DPPC/PEG-lipid LNPs (I-III; Figure 1B,C) exhibited DOTAP-dependent lung accumulation, yet severe aggregation upon nucleic acid loading rendered them unsuitable for delivery (Figure 1D). For four-component LNPs containing Dlin-MC3-DMA, an inverted U-shaped relationship emerged between the DOTAP/DPPC ratio and pulmonary fluorescence (Figure 1C), with the optimal ratio shifting based on ionizable lipid content. Notably, formulation IX (Dlin-MC3-DMA:DOTAP:DPPC:DMG-PEG2000 = 32.8:16.4:49.3:1.5) achieved 36.1% of total major-organ fluorescence in the lungs, significantly surpassing formulation VI (28.2%), while maintaining excellent colloidal stability post-nucleic acid loading (Figure 1E–G). The essential role of DPPC was confirmed by the diminished lung signal in DPPC-depleted formulations (Figure 1B,C). Based on superior targeting efficiency and oligonucleotide compatibility, formulation IX was selected as the optimal lung-targeted LNP (named as LuT-LNP; patent no. CN 202610098221X).

### 3.2. Physiological and Pathological Lung-Targeted Efficiency of LuT-LNP

**Figure 2:**
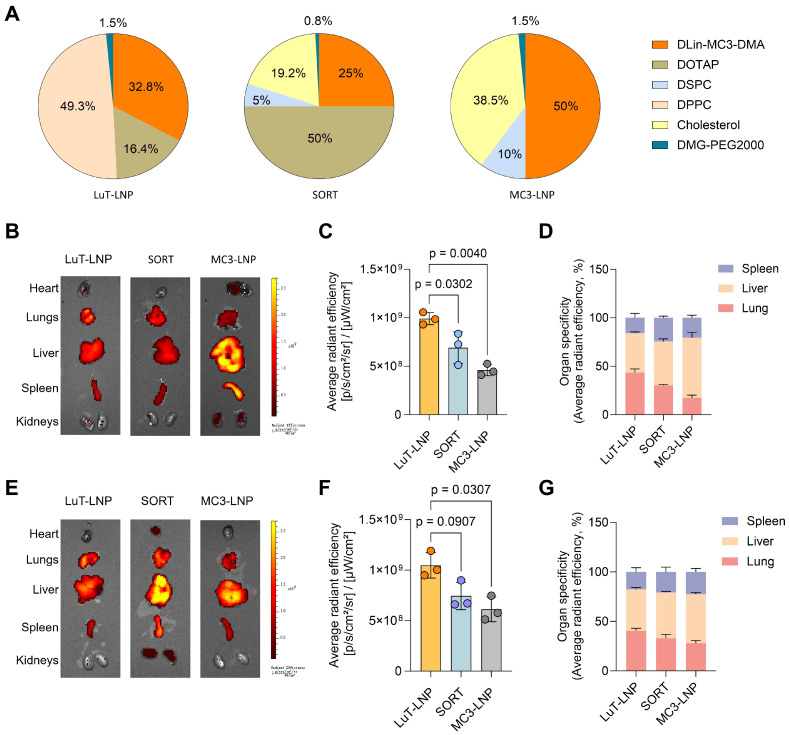
Organ distribution of LuT-LNP in normal and CRPM mouse models. (**A**) Composition of SORT and MC3-LNP formulations. (**B**) *Ex vivo* fluorescence images of organs from normal mice and (**E**) CRPM mice treated with different formulations. (**C**) Quantified fluorescence intensity in lung tissues of normal mice and (**F**) CRPM mice. (**D**,**G**) Proportion of fluorescence intensity in lungs, livers, and spleens for normal and CRPM mice, respectively. Data are presented as mean ± SD (*n* = 3).

We next compared LuT-LNP against formulations based on the SORT strategy and conventional MC3-LNP (Figure 2A). LuT-LNP demonstrated significantly higher pulmonary accumulation than both SORT-formulated and MC3-LNP systems in normal mice (Figure 2B,C). Analysis of organ fluorescence intensity ratios further confirmed that LuT-LNP increased the relative proportion of signal in the lungs (Figure 2D). To evaluate lung accumulation in a disease model, we established a CRPM model by intravenous injection of CT26-Luc cells. Consistent with findings in normal mice, LuT-LNP exhibited strong lung accumulation in CRPM-bearing mice (Figure 2E,F), with a higher lung fluorescence ratio than the SORT and MC3-LNP groups (Figure 2G). Collectively, the optimized LuT-LNP system demonstrated proficient lung-targeted efficacy in both normal and CRPM mouse models. Importantly, LuT-LNP achieved not only a high pulmonary distribution percentage but also the greatest absolute lung accumulation, a critical factor for effective oligonucleotide delivery.

### 3.3. Characterization of AM22@LuT-LNP

**Figure 3:**
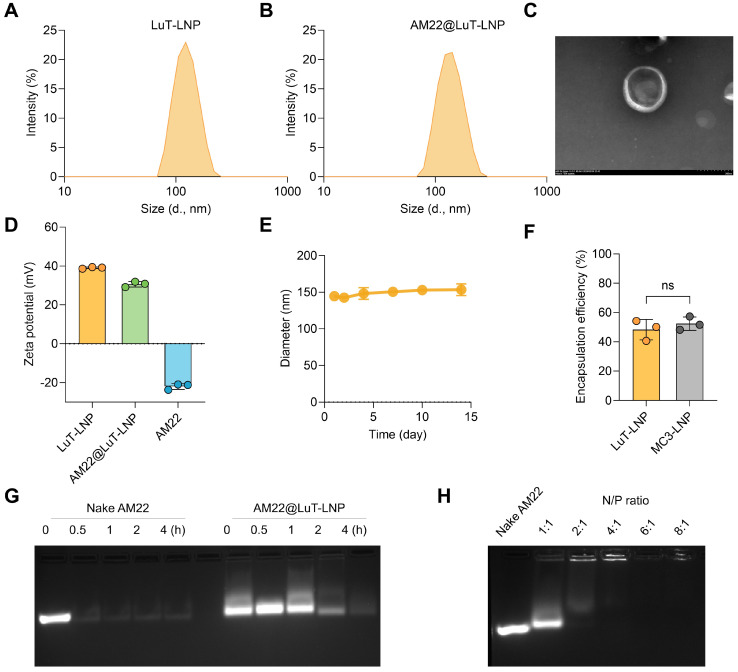
Characterizations of LuT-LNP and AM22@LuT-LNP. (**A**,**B**) Hydrodynamic size distribution of LuT-LNP and AM22@LuT-LNP. (**C**) Representative TEM image of LuT-LNP. (**D**) Zeta potential of LuT-LNP, AM22@LuT-LNP, and naked AM22. (**E**) Size stability of LuT-LNP in PBS at 4 °C over 14 days. (**F**) Encapsulation efficiency comparison between LuT-LNP and MC3-LNP. (**G**) Gel electrophoresis analysis of AM22 stability in the presence of RNase over 4 h. (**H**) Gel retardation assay evaluating AM22 encapsuled in LuT-LNP at different N/P ratios. Data are mean ± SD (*n* = 3).

AM22@LuT-LNP was prepared using the thin-film hydration method. Dynamic light scattering (Figure 3A,B) revealed that LuT-LNP had an average diameter of approximately 128 nm (PDI = 0.117 ± 0.044), which increased slightly to about 142 nm after encapsulation of AM22 (PDI = 0.159 ± 0.010). TEM confirmed a homogeneous spherical morphology of AM22@LuT-LNP (Figure 3C). The zeta potentials of LuT-LNP and AM22@LuT-LNP were approximately +40 mV and +30 mV, respectively (Figure 3D). Particle size remained virtually unchanged after 14 days of storage at 4 °C, indicating good stability (Figure 3E).

The encapsulation efficiency of LuT-LNP for nucleic acids was approximately 50%, which is comparable to that of the commercial MC3-LNP formulation (Figure 3F). Agarose gel electrophoresis assessed the protective effect of LuT-LNP against RNase degradation. Naked AM22 was almost completely degraded within 30 min, whereas AM22 encapsulated in LuT-LNP showed detectable degradation only after 4 h of exposure (Figure 3G), demonstrating effective nuclease protection. Furthermore, agarose gel retardation assays at different N/P ratios indicated complete nucleic acid encapsulation at an N/P ratio of 4:1 (Figure 3H), which was therefore selected for subsequent experiments.

### 3.4. Biosafety and Cellular Uptake of LuT-LNP

**Figure 4:**
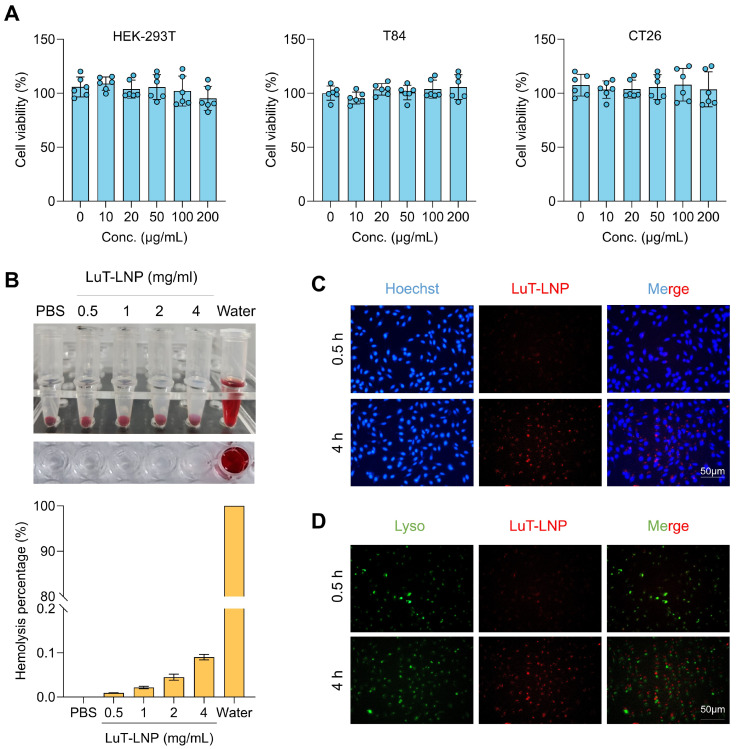
Biosafety and cellular uptake of LuT-LNP. (**A**) Cell viability of HEK-293T, T84, and CT26 cells after 72-h incubation with increasing concentrations of LuT-LNP. (**B**) Hemolysis assay of LuT-LNP. (**C**) Images showing nuclei (blue), LuT-LNPs (red), and merged channels at 0.5 and 4 h in T84 cells. (**D**) Images showing lysosomes (green), LuT-LNPs (red), and merged channels at the same time points.

The cytotoxicity of LuT-LNP was evaluated in normal (HEK-293T) and CRC (T84, CT26) cell lines using MTT assays. At concentrations up to 200 μg/mL, LuT-LNP did not significantly inhibit the viability of the cell lines (Figure 4A), indicating favorable biocompatibility. Hemolysis assays (Figure 4B) showed that LuT-LNP induced negligible hemolysis (<0.1%) across all tested concentrations, further confirming its blood compatibility.

Cellular uptake and intracellular trafficking were visualized by inverted fluorescent microscope in T84 cells. LuT-LNP was labeled with DiR (red), nuclei with Hoechst 33,342 (blue), and lysosomes with a green fluorescent probe. The red fluorescence signal was weak at 0.5 h but markedly increased by 4 h, confirming time-dependent cellular uptake (Figure 4C). Notably, the red fluorescence showed minimal overlap with green lysosomal signal at 4 h, suggesting efficient endolysosomal escape, a desirable feature for prolonged intracellular activity (Figure 4D).

### 3.5. In Vitro Anti-Tumor Activity of AM22@LuT-LNP

**Figure 5:**
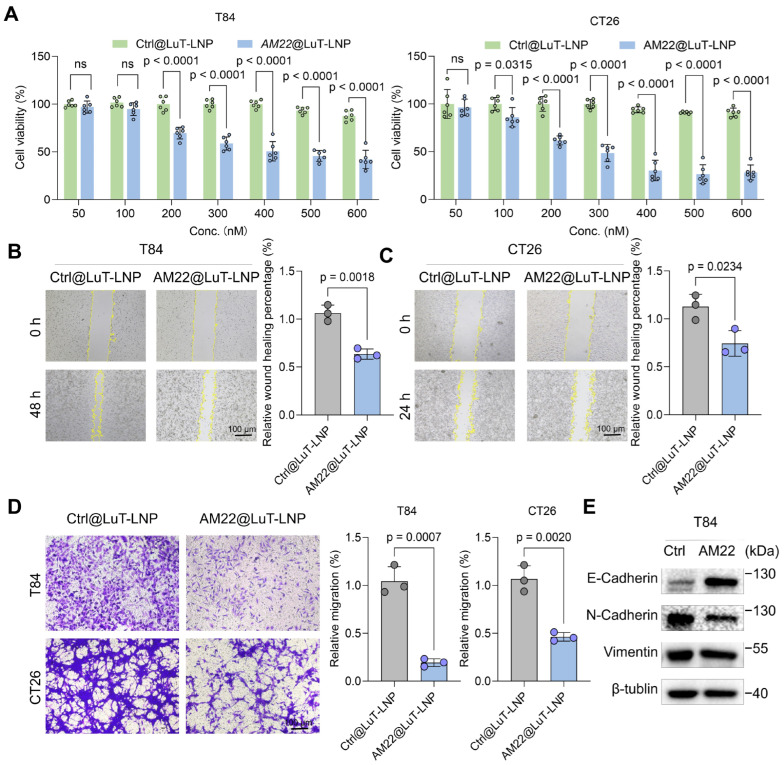
AM22@LuT-LNP inhibits proliferation and migration of CRC cell lines. (**A**) Proliferation inhibition in T84 and CT26 cells treated with AM22@LuT-LNP or Ctrl@LuT-LNP. (**B**,**C**) Wound-healing assays quantifying migration of T84 and CT26 cells upon the treatment of AM22@LuT-LNP or Ctrl@LuT-LNP. (**D**) Transwell migration assays for T84 and CT26 cells upon the treatment of AM22@LuT-LNP or Ctrl@LuT-LNP. (**E**) Western blot analysis of EMT-related protein expression in T84 cells after treatment of AM22@LuT-LNP or Ctrl@LuT-LNP. Data are mean ± SD (*n* = 3).

The anti-tumor efficacy of AM22@LuT-LNP was assessed in human metastatic T84 and murine CT26 CRC cells. Proliferation assays revealed that AM22@LuT-LNP suppressed the cell proliferation in a concentration-dependent manner, with significant inhibition at higher concentrations, while Ctrl@LuT-LNP (loaded with non-functional miRNA) had minimal effect on cell viability (Figure 5A). Wound-healing assays demonstrated that AM22@LuT-LNP significantly reduced the migration rate and relative wound closure area in both T84 and CT26 cells compared with the Ctrl@LuT-LNP group (Figure 5B,C). Similarly, Transwell migration assays showed a marked decrease in the number of migrated cells following AM22@LuT-LNP treatment (Figure 5D). Western blot analysis further indicated that AM22 treatment upregulated E-cadherin and downregulated N-cadherin expression (Figure 5E), suggesting inhibition of EMT. Collectively, these findings demonstrate that AM22@LuT-LNP effectively impairs the proliferative and migratory capacities of CRC cells *in vitro*.

### 3.6. Anti-Tumor Activity of AM22@LuT-LNP in CRPM Mice

**Figure 6:**
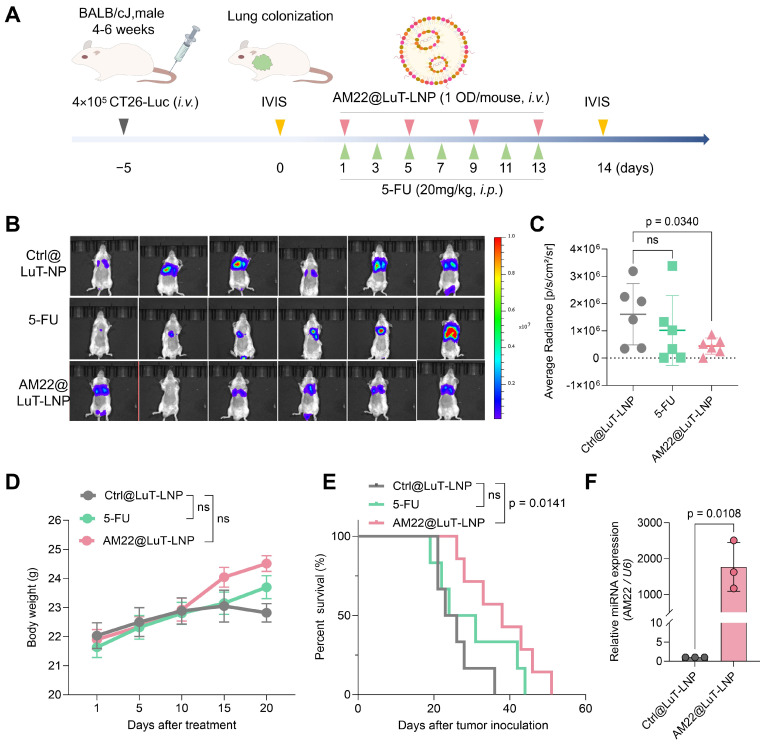
AM22@LuT-LNP delays tumor progression in mice. (**A**) Experimental timeline and treatment schedule. (**B**) *In vivo* bioluminescence images and (**C**) quantified fluorescence intensity of mice after treatment with 5-FU, Ctrl@LuT-LNP, or AM22@LuT-LNP (*n* = 6). (**D**) Body weight changes of mice during treatment. (**E**) Survival curves of the three treatment groups. (**F**) Relative miRNA expression level of AM22 in the lung tissues of CRPM mice (*n* = 3). Data are mean ± SD.

The *in vivo* anti-tumor efficacy of AM22@LuT-LNP was evaluated in a CRPM mouse model established via tail-vein injection of CT26-Luc cells. Mice received either 5-FU (positive control), Ctrl@LuT-LNP, or AM22@LuT-LNP according to the schedule in Figure 6A. Bioluminescence imaging revealed rapid tumor progression in the Ctrl@LuT-LNP group and variable progression in the 5-FU group, whereas the AM22@LuT-LNP group showed significantly suppressed lung luminescence (Figure 6B,C), indicating effective tumor growth inhibition. No significant body weight loss was observed in any group (Figure 6D), demonstrating the treatments are safe. Moreover, AM22@LuT-LNP treatment significantly extended the survival of CRPM-bearing mice compared with the control group (Figure 6E). Notably, high level of AM22 was detected in the lungs of CRPM mice after treatment of AM22@LuT-LNP (Figure 6F), which further confirms efficient lung-targeted delivery of AM22 by LuT-LNP.

### 3.7. Anti-Tumor Mechanism of AM22@LuT-LNP

**Figure 7:**
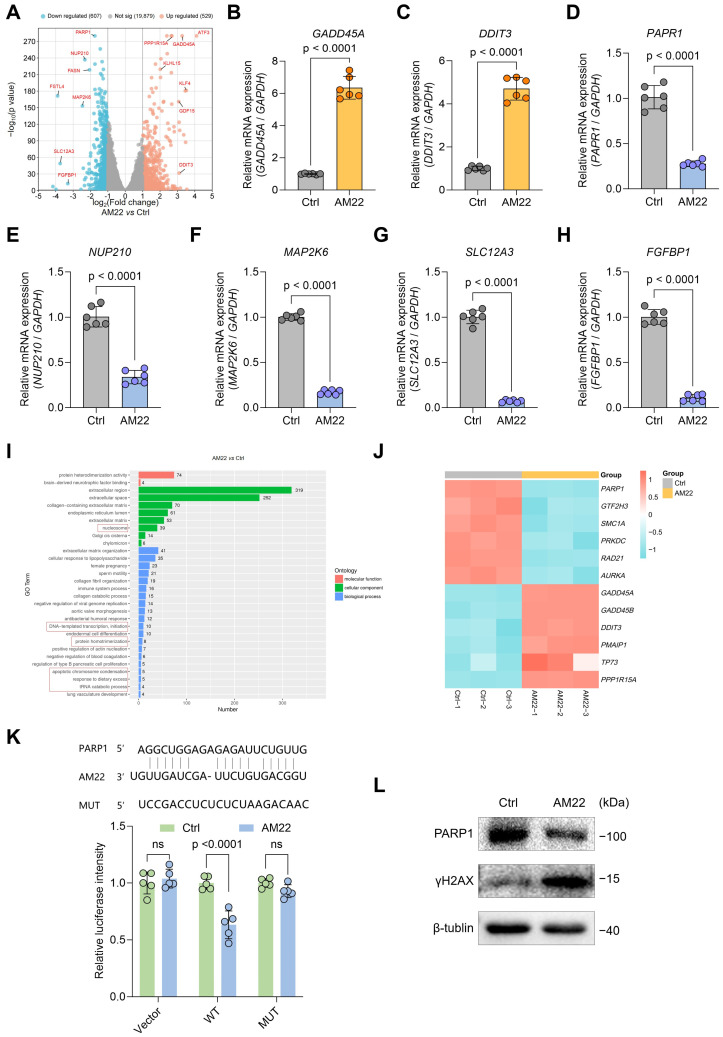
Anti-tumor mechanisms of AM22@LuT-LNP against CRPM. (**A**) Volcano plot of DEGs. (**B**–**H**) qPCR validation of the indicated DEGs. (**I**) Gene Ontology enrichment of DEGs. (**J**) Heatmap of DEGs related to DNA damage. (**K**) Dual-luciferase reporter assay confirming *PARP1* as a direct target of AM22. (**L**) The impact of AM22@LuT-LNP on PARP1 and γ-H2AX expression. Data are mean ± SD (*n* = 6).

To investigate the anti-tumor mechanisms of AM22@LuT-LNP, we performed RNA-seq on T84 cells treated with AM22@LuT-LNP. Differential expression analysis revealed 1136 significantly altered genes, including 529 upregulated and 607 downregulated transcripts as compared with Ctrl@LuT-LNP. Among them, cancer-associated genes such as *PARP1*, *NUP210*, *MAP2K6*, *SLC12A3*, and *FGFBP1* were markedly downregulated, whereas *GADD45A* and *DDIT3* were significantly upregulated (Figure 7A), which were further confirmed by qPCR validation (Figure 7B–H, Table 1). Gene Ontology enrichment analysis demonstrated that differentially expressed genes (DEGs) were prominently enriched in pathways related to apoptotic chromosome condensation, nucleosome organization, and DNA transcriptional regulation, indicating that AM22 mainly modulated the DNA damage response and cell apoptosis (Figure 7I). The heatmap visualization of DNA damage-related genes showed that AM22 treatment markedly suppressed genes involved in DNA damage repair including *PARP1*, *PRKDC*, *RAD21*, *SMC1A*, *GTF2H3*, and *AURKA*, and substantially induced genes associated with DNA damage sensing and apoptosis, such as *GADD45A*, *GADD45B*, *DDIT3*, *PMAIP1*, *TP73*, and *PPP1R15A* (Figure 7J). Subsequent dual-luciferase reporter assays confirmed that AM22 specifically binds to the wild-type but not the mutant 3′-UTR of *PARP1* mRNA (Figure 7K), indicating *PARP1* is a direct molecular target of AM22. In line with this, western blot analysis demonstrated that AM22 treatment obviously reduced PARP1 protein levels and increased the abundance of the DNA damage marker γ-H2AX in T84 cells (Figure 7L).

## 4. Discussion

In this study, we successfully developed a novel lung-targeted lipid nanoparticle (LuT-LNP) platform for the efficient delivery of the tumor-suppressive miRNA AM22, aiming to suppress CRPM. By replacing conventional cholesterol and helper phospholipids with DPPC, the predominant phospholipid in pulmonary surfactant, and incorporating the permanently cationic lipid DOTAP, we substantially enhanced the lung tropism of the LNPs. The optimized LuT-LNP formulation exhibited favorable colloidal stability, biocompatibility, and nucleic acid encapsulation efficiency, along with potent anti-tumor activity and robust lung-specific accumulation both *in vitro* and *in vivo*.

The realization of therapeutic nucleic acid efficacy is critically dependent on the development of effective delivery systems. Recent advances in lung-targeted delivery encompass diverse strategies, including ligand–drug conjugates [26], spray-dried formulations for respiratory diseases [27], and engineered nanoparticles [28]. Among these, LNPs represent a particularly promising platform owing to their high payload capacity, scalable manufacturing, and favorable safety profile, rendering them ideal carriers for nucleic acid therapeutics. Here, we specifically selected the LNP delivery platform not only for the general advantages mentioned above, but also for its modular lipid composition. This design allows us to directly replace the cholesterol and helper lipids in conventional LNPs with DPPC, the most abundant phospholipid in pulmonary surfactant, while incorporating DOTAP. This rational design confers lung tropism to the LNPs without the need for chemically complex or non-biodegradable materials, representing a distinct advantage over conventional lung-targeting strategies that often rely on chemical conjugation. Building upon prior SORT strategies and related lung-directed designs, we performed comprehensive *in vivo* biodistribution screening of formulations with various lipid compositions and ratios, and identified LuT-LNP, which demonstrated superior lung accumulation compared with established benchmark formulations.

Although the detailed molecular mechanism underlying the lung-targeting property of LuT-LNP remains to be fully elucidated, we propose a plausible explanation based on current knowledge and our observations. The lung tropism of LuT-LNP is likely driven by the combined contributions of its two key lipid components. On the one hand, DPPC, the most abundant phospholipid in pulmonary surfactant, presents on the particle surface may exhibit intrinsic affinity for pulmonary surfactant components or lung capillary endothelial cells, thereby facilitating nanoparticle retention and accumulation in the lungs. On the other hand, the incorporated DOTAP is hypothesized to mediate lung targeting through an endogenous pathway similar to the SORT mechanism that gradual desorption of PEG lipids exposes DOTAP and recruits a protein corona enriched in vitronectin; vitronectin subsequently binds to integrin receptors that are highly expressed on lung endothelial cells, driving preferential pulmonary distribution. Together, these DPPC- and DOTAP-dependent processes provide a rational basis for the superior lung accumulation of LuT-LNP.

As a therapeutic paradigm succeeding small molecules and antibodies, nucleic acid-based therapy has demonstrated unique efficacy in rare and chronic diseases and holds considerable promise in oncology [29]. AM22, a miRNA derivative previously engineered via nucleotide substitution within the non-seed region of miR-34a, has exhibited potent anti-tumor activity in cellular models and subcutaneous xenografts [16]. In the current study, the AM22@LuT-LNP system significantly suppressed the proliferation and migration of CRC cells *in vitro* and exerted pronounced anti-metastatic effects in a murine CRPM model. Mechanistic investigation revealed that multiple differentially expressed genes (DEGs) were intimately associated with DNA damage response pathways. Notably, *PARP1*, which encodes a critical DNA repair enzyme frequently dysregulated in malignancies, represents a validated therapeutic target in ovarian and breast cancers (e.g., via olaparib) [30]. *GADD45A*, a stress-responsive gene induced upon DNA damage, functions as a tumor suppressor and orchestrates stress signaling [31,32]. As a transcription factor induced to express during DNA damage, *DDIT3* mainly participates in the repair or clearance of damaged cells by regulating cell cycle arrest and apoptosis [33]. Importantly, we identified *PARP1* as a direct molecular target of AM22. AM22 binds specifically to the 3′-UTR of *PARP1* mRNA, repressing its expression, triggering DNA damage accumulation, and inhibiting EMT. This finding not only links AM22 directly to the DNA damage response and key metastatic pathways, thereby deepening our understanding of its anti-tumor mechanisms, but also provides a compelling rationale for potential combination therapies with PARP inhibitors or other DNA damage-targeting agents.

Despite these promising results, certain limitations remain. First, the precise mechanism underlying the lung targeting of LuT-LNP requires further elucidation. Specifically, the interactions between DPPC and pulmonary surfactant components, as well as the detailed pathways by which DOTAP mediates targeting to lung endothelial cells, warrant deeper investigation. Further experiments, such as surface-active protein-binding assays and analysis of lung endothelial cell uptake pathways, are also required to elucidate the underlying mechanism. Second, the current validation was performed in transplanted tumor models; the long-term efficacy and safety of this system should be further assessed in spontaneous metastasis or patient-derived xenograft models. Additionally, potential off-target effects of AM22 and its impact on normal lung tissue need systematic evaluation. Finally, exploring combination strategies of LuT-LNP with immunotherapy, chemotherapy, or targeted therapies may offer more comprehensive and effective treatment options for CRPM and other pulmonary metastatic cancers.

## 5. Conclusions

In summary, this study establishes a lung-targeted lipid nanoparticle platform, LuT-LNP, that enables efficient pulmonary delivery of the tumor-suppressive miRNA AM22 for the treatment of CRPM. By rationally substituting conventional LNP components with DPPC and systematically optimizing the lipid composition, we achieved superior lung tropism and robust nucleic acid protection. The AM22@LuT-LNP formulation potently inhibited CRC cell proliferation and migration *in vitro* and significantly suppressed metastatic progression *in vivo* without evident systemic toxicity. Mechanistically, we identified *PARP1* as a direct target of AM22, linking AM22-mediated gene silencing to DNA damage accumulation and suppression of EMT. Collectively, these findings not only provide a promising lung-targeted delivery strategy for miRNA-based cancer therapy but also offer mechanistic rationale for future combination regimens targeting DNA repair pathways in metastatic CRC.

## Figures and Tables

**Table 1:** PCR and qPCR primer sequences.

Gene	Forward Primer (5′-3′)	Reverse Primer (5′-3′)
AM22	CGCGTGGCAGTGTCTTAGCT	AGTGCAGGGTCCGAGGTATT
*DDIT3*	GGAAACAGAGTGGTCATTCCC	CTGCTTGAGCCGTTCATTCTC
*FGFBP1*	GGAAACAAGTTGCCCGGAATC	AATAGAGTGGAGCTGACTAGCTT
*GADD45A*	GAGAGCAGAAGACCGAAAGGA	CAGTGATCGTGCGCTGACT
*GAPDH*	CTGGGCTACACTGAGCACC	AAGTGGTCGTTGAGGGCAATG
*MAP2K6*	GAAGCATTTGAACAACCTCAGAC	CCTGGCTATTTACTGTGGCTC
*NUP210*	GCACTATCTACGTGGTCGAAC	CCTCGAAGAACTCAGCAGGAA
*PARP1*	CGGAGTCTTCGGATAAGCTCT	TTTCCATCAAACATGGGCGAC
*SLC12A3*	CCTGGGTGGAGACCTTCATTC	GAGCCCCAATTTACCTCTGGC

## Data Availability

The original contributions presented in this study are included in the article. Further inquiries can be directed to the corresponding authors.

## References

[1] Siegel R.L., Kratzer T.B., Giaquinto A.N., Sung H., Jemal A. (2025). Cancer statistics, 2025. CA Cancer J. Clin..

[2] Bray F., Laversanne M., Sung H., Ferlay J., Siegel R.L., Soerjomataram I., Jemal A. (2024). Global cancer statistics 2022: GLOBOCAN estimates of incidence and mortality worldwide for 36 cancers in 185 countries. CA Cancer J. Clin..

[3] Akimoto N., Ugai T., Zhong R., Hamada T., Fujiyoshi K., Giannakis M., Wu K., Cao Y., Ng K., Ogino S. (2021). Rising incidence of early-onset colorectal cancer―A call to action. Nat. Rev. Clin. Oncol..

[4] Morgan E., Arnold M., Gini A., Lorenzoni V., Cabasag C.J., Laversanne M., Vignat J., Ferlay J., Murphy N., Bray F. (2023). Global burden of colorectal cancer in 2020 and 2040: Incidence and mortality estimates from GLOBOCAN. Gut.

[5] Eng C., Yoshino T., Ruiz-Garcia E., Mostafa N., Cann C.G., O’Brian B., Benny A., Perez R.O., Cremolini C. (2024). Colorectal cancer. Lancet.

[6] André T., Boni C., Mounedji-Boudiaf L., Navarro M., Tabernero J., Hickish T., Topham C., Zaninelli M., Clingan P., Bridgewater J. (2004). Oxaliplatin, fluorouracil, and leucovorin as adjuvant treatment for colon cancer. N. Engl. J. Med..

[7] Le D.T., Durham J.N., Smith K.N., Wang H., Bartlett B.R., Aulakh L.K., Lu S., Kemberling H., Wilt C., Luber B.S. (2017). Mismatch repair deficiency predicts response of solid tumors to PD-1 blockade. Science.

[8] André T., Shiu K.K., Kim T.W., Jensen B.V., Jensen L.H., Punt C., Smith D., Garcia-Carbonero R., Benavides M., Gibbs P. (2020). Pembrolizumab in Microsatellite-Instability-High Advanced Colorectal Cancer. N. Engl. J. Med..

[9] Williams C.J., Peddle A.M., Kasi P.M., Seligmann J.F., Roxburgh C.S., Middleton G.W., Tejpar S. (2024). Neoadjuvant immunotherapy for dMMR and pMMR colorectal cancers: Therapeutic strategies and putative biomarkers of response. Nat. Rev. Clin. Oncol..

[10] Emiloju O.E., Sinicrope F.A. (2023). Neoadjuvant Immune Checkpoint Inhibitor Therapy for Localized Deficient Mismatch Repair Colorectal Cancer: A Review. JAMA Oncol..

[11] Lv X., Sun X., Gao Y., Song X., Hu X., Gong L., Han L., He M., Wei M. (2025). Targeting RNA splicing modulation: New perspectives for anticancer strategy?. J. Exp. Clin. Cancer Res..

[12] Elbashir S.M., Harborth J., Lendeckel W., Yalcin A., Weber K., Tuschl T. (2001). Duplexes of 21-nucleotide RNAs mediate RNA interference in cultured mammalian cells. Nature.

[13] Kim D.H., Behlke M.A., Rose S.D., Chang M.S., Choi S., Rossi J.J. (2005). Synthetic dsRNA Dicer substrates enhance RNAi potency and efficacy. Nat. Biotechnol..

[14] Zong Y., Lin Y., Wei T., Cheng Q. (2023). Lipid Nanoparticle (LNP) Enables mRNA Delivery for Cancer Therapy. Adv. Mater..

[15] Liu C., Shi Q., Huang X., Koo S., Kong N., Tao W. (2023). mRNA-based cancer therapeutics. Nat. Rev. Cancer.

[16] Meng F., Li J., Qiu Y., Zhang H., Zhang H., Wang W. (2022). AM22, a novel synthetic microRNA, inhibits the proliferation of colorectal cancer cells by targeting core binding factor subunit beta (CBFB). Investig. New Drugs.

[17] Tang Q., Khvorova A. (2024). RNAi-based drug design: Considerations and future directions. Nat. Rev. Drug Discov..

[18] Yu A.M., Tu M.J. (2022). Deliver the promise: RNAs as a new class of molecular entities for therapy and vaccination. Pharmacol. Ther..

[19] Adams D., Gonzalez-Duarte A., O’Riordan W.D., Yang C.C., Ueda M., Kristen A.V., Tournev I., Schmidt H.H., Coelho T., Berk J.L. (2018). Patisiran, an RNAi Therapeutic, for Hereditary Transthyretin Amyloidosis. N. Engl. J. Med..

[20] Zhang Y., Sun C., Wang C., Jankovic K.E., Dong Y. (2021). Lipids and Lipid Derivatives for RNA Delivery. Chem. Rev..

[21] Eygeris Y., Gupta M., Kim J., Sahay G. (2022). Chemistry of Lipid Nanoparticles for RNA Delivery. Acc. Chem. Res..

[22] Cullis P.R., Felgner P.L. (2024). The 60-year evolution of lipid nanoparticles for nucleic acid delivery. Nat. Rev. Drug Discov..

[23] Cheng Q., Wei T., Farbiak L., Johnson L.T., Dilliard S.A., Siegwart D.J. (2020). Selective organ targeting (SORT) nanoparticles for tissue-specific mRNA delivery and CRISPR-Cas gene editing. Nat. Nanotechnol..

[24] Su K., Shi L., Sheng T., Yan X., Lin L., Meng C., Wu S., Chen Y., Zhang Y., Wang C. (2024). Reformulating lipid nanoparticles for organ-targeted mRNA accumulation and translation. Nat. Commun..

[25] Qiu M., Tang Y., Chen J., Muriph R., Ye Z., Huang C., Evans J., Henske E.P., Xu Q. (2022). Lung-selective mRNA delivery of synthetic lipid nanoparticles for the treatment of pulmonary lymphangioleiomyomatosis. Proc. Natl. Acad. Sci. USA.

[26] Pastor F., Berraondo P., Etxeberria I., Frederick J., Sahin U., Gilboa E., Melero I. (2018). An RNA toolbox for cancer immunotherapy. Nat. Rev. Drug Discov..

[27] Zimmermann C.M., Baldassi D., Chan K., Adams N.B., Neumann A., Porras-Gonzalez D.L., Wei X., Kneidinger N., Stoleriu M.G., Burgstaller G. (2022). Spray drying siRNA-lipid nanoparticles for dry powder pulmonary delivery. J. Control. Release.

[28] Kranz L.M., Diken M., Haas H., Kreiter S., Loquai C., Reuter K.C., Meng M., Fritz D., Vascotto F., Hefesha H. (2016). Systemic RNA delivery to dendritic cells exploits antiviral defence for cancer immunotherapy. Nature.

[29] Sun X., Setrerrahmane S., Li C., Hu J., Xu H. (2024). Nucleic acid drugs: Recent progress and future perspectives. Signal Transduct. Target. Ther..

[30] Chappidi N., Quail T., Doll S., Vogel L.T., Aleksandrov R., Felekyan S., Kühnemuth R., Stoynov S., Seidel C.A., Brugués J. (2024). PARP1-DNA co-condensation drives DNA repair site assembly to prevent disjunction of broken DNA ends. Cell.

[31] Barreto G., Schäfer A., Marhold J., Stach D., Swaminathan S.K., Handa V., Döderlein G., Maltry N., Wu W., Lyko F. (2007). *Gadd45a* promotes epigenetic gene activation by repair-mediated DNA demethylation. Nature.

[32] Hollander M.C., Sheikh M.S., Bulavin D.V., Lundgren K., Augeri-Henmueller L., Shehee R., Molinaro T.A., Kim K.E., Tolosa E., Ashwell J.D. (1999). Genomic instability in *Gadd45a*-deficient mice. Nat. Genet..

[33] Ni R., Cao T., Ji X., Peng A., Zhang Z., Fan G.C., Stathopulos P., Chakrabarti S., Su Z., Peng T. (2025). DNA damage-inducible transcript 3 positively regulates RIPK1-mediated necroptosis. Cell Death Differ..

